# White Matter fMRI Activation Cannot Be Treated as a Nuisance Regressor: Overcoming a Historical Blind Spot

**DOI:** 10.3389/fnins.2019.01024

**Published:** 2019-10-04

**Authors:** Lukas A. Grajauskas, Tory Frizzell, Xiaowei Song, Ryan C. N. D’Arcy

**Affiliations:** ^1^Department of Biomedical Physiology and Kinesiology, Simon Fraser University, Burnaby, BC, Canada; ^2^Cumming School of Medicine, University of Calgary, Calgary, AB, Canada; ^3^ImageTech Lab, Surrey Memorial Hospital, Fraser Health, Surrey, BC, Canada; ^4^Faculty of Applied Sciences, Simon Fraser University, Burnaby, BC, Canada; ^5^Djavad Mowafaghian Centre for Brain Health, University of British Columbia, Vancouver, BC, Canada

**Keywords:** white matter, BOLD, fMRI, motion regression, fMRI analysis

## Abstract

Despite past controversies, increasing evidence has led to acceptance that white matter activity is detectable using functional magnetic resonance imaging (fMRI). In spite of this, advanced analytic methods continue to be published that reinforce a historic bias against white matter activation by using it as a nuisance regressor. It is important that contemporary analyses overcome this blind spot in whole brain functional imaging, both to ensure that newly developed noise regression techniques are accurate, and to ensure that white matter, a vital and understudied part of the brain, is not ignored in functional neuroimaging studies.

## Introduction

White matter is a vital part of the human brain, but in functional magnetic resonance imaging (fMRI), it remains largely overlooked and misunderstood. More than 15 years of evidence has shown that neural activation occurring in white matter can be detected using blood-oxygenation level dependent (BOLD) fMRI, and the existing literature has been well reviewed by Gawryluk et al. (2014b) and Gore et al. (2019). Despite this, white matter activation in fMRI is largely under-represented and a great deal of research even ignores this evidence entirely and treats white matter in an inappropriate manner. Operating on the outdated assumption that white matter BOLD signal has no physiological component, many researchers have used the signal from white matter as a “nuisance regressor” to remove noise from fMRI signals. The evidence showing that physiological signals are present means the use of this technique is incorrect, however, these approaches continue to be employed (e.g., Jo et al., 2013; Power et al., 2014; Ciric et al., 2017). Several recent papers that used white matter as a nuisance regressor even cited literature supporting the existence of physiological signals in white matter, but then proceeded regardless of this evidence, offering little in the form of counterargument. For example, Bartoň et al. (2019) acknowledged the existence of fMRI detectable white matter activation, but then continued to use white matter signals as a nuisance regressor, with no justification or rationale. Similarly, Yang et al. (2019) acknowledged some of the white matter activation literature, but then also disregarded it, citing the “lack of neurons in white matter,” a statement that is clearly anatomically incorrect (Kukley et al., 2007; García-Marín et al., 2010). These examples and others like them underscore the blind spot that has been carried into even the most contemporary fMRI research. The field needs to update its understanding and give appropriate credence to the evidence for detectable white matter BOLD signals in fMRI.

## Physiological Precedent

The belief that white matter activation cannot be detected has become engrained in field of fMRI research, but this idea is not based on fundamental physiological principles. Instead, researchers have come to this belief simply because of a lack of reports in the literature. However, this absence may have simply stemmed from the difficulty of detecting the lower magnitude white matter BOLD signals on early 1.5T scanners, as field strength has been shown to play a large role in the detection of white matter activity (Mazerolle et al., 2013; Gawryluk et al., 2014b). Despite this, there is physiological evidence that suggests a BOLD response can be detected in white matter, albeit one that is smaller and harder to detect. While our traditional understanding is that the BOLD response is primarily driven by the high energy demands of synaptic transmission, there is evidence that action potentials alone could drive a BOLD response. Histology indicates there is vasculature in white matter (Lierse and Horstmann, 1965; Duvernoy et al., 1981), and that this vasculature stems largely from the medullary artery which has no vascular interaction with cortical tissue (Nonaka et al., 2003; Akashi et al., 2017), meaning the energy demands of gray matter will not influence its oxygenation. The axons in white matter have been shown to be metabolically active (Takii et al., 2003), containing mitochondria all along their length (Misgeld et al., 2007), which require oxygen to produce ATP for cellular processes such as the re-establishment of ionic gradients after the transmission of action potentials. Oxygen uptake in isolated axons has been shown to be modified by changes in Na-/K+ pump activity (Hargittai et al., 1987), and oxygen demand increases in axons undergoing repeated action potentials (Ritchie, 1967). Furthermore, a linkage between active axons and glia has been shown (Hargittai and Lieberman, 1991), where glia will activate potassium pumps to help restore ionic balance during an action potential (Petzold and Murthy, 2011) and therefore require oxygen in a task dependent manner. Overall, the evidence to support task dependent hemodynamic changes in white matter coupled with accumulating evidence from imaging should cause researchers to rethink their assumptions about white matter fMRI.

## Reports of fMri Activation in White Matter

Alongside this physiological precedent, more than 15 years of fMRI evidence has been published to support the existence of a detectable white matter BOLD signal, across a range of different tasks. One of the most robust methods for detection of white matter BOLD is using an “interhemispheric transfer task” to drive communication between hemispheres. This has consistently elicited detectible activation in the corpus callosum (Tettamanti et al., 2002; Omura et al., 2004; Weber et al., 2005; D’Arcy et al., 2006; Mazerolle et al., 2008; Gawryluk et al., 2009, 2011a). Mazerolle et al. (2010) further combined DTI with white matter BOLD to show that activated areas in the corpus callosum were structurally connected to the cortical areas activated by the interhemispheric transfer task. Fabri et al. (2011) also showed distinct patterns of activation in the corpus callosum for tactile, gustatory, visual, and motor tasks. Activation has also been shown in the corticospinal tract during swallowing (Mosier et al., 1999) and during a finger tapping task (Gawryluk et al., 2011b; Mazerolle et al., 2013), in the optic radiations during a visual task (Brandt et al., 2000), and in the internal capsule during a symbol digit modalities test (Gawryluk et al., 2014a). Weis et al. (2011) also found white matter activation during a memory task in both healthy controls and Alzheimer’s patients. White matter activation has also been studied in resting state MRI, for example Ding et al. (2013) showed that functional connectivity within white matter tracts in the corpus callosum and optic radiations was greater within the tract than when compared with other voxels matched in distance from the seed. Ding et al. (2018) also used resting state MRI to show that there was connectivity between cortical regions and specific white matter tracts. This group was also able to create “functional connectivity tensors” similar to diffusion tensors by assessing the degree of signal correlation between a voxel and its adjacent neighbors, and representing the directionality of this correlation as a tensor (Ding et al., 2016). These functional correlation tensors present an interesting new methodology for investigating white matter activity and connectivity within the brain. Overall, these studies have taken great care to rule out partial volume effects (Gawryluk et al., 2014b), and the correspondence between the nature of the task and the white matter tract activated gives credence to the idea that this represents real activation, not simply artifact.

To add to this, recently published work has also made it clear that white matter has a different hemodynamic profile than the models that have been traditionally used in gray matter. A recent paper focused on characterizing the hemodynamic response function (HRF) at different depths of white matter was published in Nature Communications (Li et al., 2019), showing that the HRFs differed significantly from gray matter, and even varied within the white matter based on the depth of the tract. Importantly, the authors confirmed prior work by Courtemanche et al. (2018) which evaluated the differences between gray matter and white matter HRFs. The differences in HRFs between gray matter and white matter also may have contributed to the lack of reports of white matter BOLD activation, as analysis techniques using the traditional gray matter HRF would not effectively capture the hemodynamic profile of white matter, further perpetuating the blind spot surrounding white matter BOLD. A number of new MRI techniques are promising for the detection of white matter BOLD, but at the very least, researchers should make use of a white matter specific HFR.

## New Tools for Better Detection of White Matter Bold fMri

The increasing evidence and acceptance surrounding white matter BOLD fMRI is well timed, as a number of technological advances allow for the better detection of white matter activation. Increases in spatial resolution will allow for the isolation of signals coming from small white matter tracts, and increases in temporal resolution will allow for better modeling of white matter hemodynamic responses and better characterization of resting state correlations. The increasing accessibility of ultra-high field scanners (7T and above) will allow for investigations with high spatial resolution, as well as improved signal- and contrast- to-noise ratios, allowing for enhanced detection and characterization of white matter signals. Multiband excitation, a technique which optimizes tradeoffs related to spatial resolution in shorter time limits, is more available (Poser and Setsompop, 2018), and high-density channel coils have also become more common, further increasing spatial and temporal resolution through parallel imaging (Hardy et al., 2008). Additionally, new developments in other neuroimaging modalities such as magnetoencephalography will allow for multi-modal investigations of white matter function (Papadelis et al., 2012; Yoshida et al., 2017). All of these techniques are already challenging previously established limits in functional neuroimaging (Feinberg and Setsompop, 2013; Petridou et al., 2013), and will have a particularly large impact on the characterization of white matter signals.

## Discussion

White matter represents a major component of functional neural tissue and plays a critical role in neural networks. Despite this, white matter activation has become a blind spot in fMRI research. Because it could not be easily detected in the early development stages of fMRI, an assumption developed that white matter activation did not exist or was not detectable. This assumption led to a self-fulfilling prophecy as analysis methods were designed to prioritize the detection of gray matter activation. Given the state of the field and the clear evidence of white matter activation, these assumptions need to be revisited otherwise the researchers will continue to proceed with a blind spot that encompasses approximately 50% of the functional neural tissue in the brain. At a minimum, fMRI analysis methodology is best to restrict the choice of nuisance regressors to non-neural tissue, such as CSF. While this paper offers a caution, it also has a more exciting perspective. Accepting the existence of detectable white matter activation opens up a host of new research questions, such as altered white matter function in injury or disease states, direct measures of functional connectivity in neural networks, and neuroplasticity changes at the network level. With increasing temporal and spatial resolution in modern MRIs, expanding our research field to include white matter activation has become readily accessible. All it will take to open up an exciting new chapter in fMRI research is for scientists to move past this outdated blind spot, and begin answering new questions using white matter BOLD fMRI.

## Author Contributions

LG contributed as primary author. TF assisted in writing the manuscript. XS and RD’A assisted in editing the manuscript and acted as lab PI.

## Conflict of Interest

The authors declare that the research was conducted in the absence of any commercial or financial relationships that could be construed as a potential conflict of interest.
